# Defining “FGF21 Resistance” during obesity: Controversy, criteria and unresolved questions

**DOI:** 10.12688/f1000research.14117.1

**Published:** 2018-03-07

**Authors:** Kathleen R. Markan

**Affiliations:** 1 Department of Pharmacology, Carver College of Medicine , University of Iowa, Iowa City, IA, 52242, USA; 2 Fraternal Order of Eagles Diabetes Research Center , University of Iowa, Iowa City, IA, 52242, USA

**Keywords:** FGF21 resistance, fibroblast growth factor 21, obesity, insulin senstivity, FGF21, beta-klotho, insulin resistance

## Abstract

The term “FGF21 resistance” was first used to describe increased circulating FGF21 levels concomitant to decreased FGF21 receptor complex expression in white adipose tissue of obese mice.  Since this initial report, the term has been associated with a wide range of pathological states, including human obesity, in which circulating FGF21 levels are elevated. However, the notion of “FGF21 resistance” has been controversial partly due to difficulty in delineating the mechanisms underlying the physiological versus pharmacological effects of FGF21.  Here, key aspects of the term “FGF21 resistance” are discussed including; the origin and experimental context surrounding the term “FGF21 resistance”, new criteria for evaluating FGF21 sensitivity *in vivo* and finally, crucial unresolved questions regarding the function of FGF21 during obesity.

Fibroblast Growth Factor 21 (FGF21) has pleiotropic metabolic effects including increasing insulin sensitivity and energy expenditure, while decreasing body weight and sugar intake ^1, 2^. Paradoxical to these beneficial metabolic effects, circulating FGF21 is increased during obesity potentially suggesting a state of “FGF21 resistance” ^3^. This hypothesis, however, has generated controversy within the field. In light of this and in response to a recent call for a unified definition of “FGF21 resistance” ^4^, I would like to discuss the controversy surrounding “FGF21 resistance” during obesity, highlight unresolved questions and outline additional criteria for its definition.

“FGF21 resistance” was first used to describe decreased expression of the FGF21 receptor complex in epididymal white adipose tissue, increased plasma FGF21, blunted ERK phosphorylation, and attenuated reduction in plasma glucose following low dose administration of FGF21 that occurred in obese mice ^3^. Shortly thereafter, an independent group also reported decreased FGF21 co-receptor expression in white adipose tissue and increased plasma FGF21 levels in obese mice ^5^. However, based on dose response studies, these investigators concluded that circulating FGF21 is increased during obesity to maintain insulin sensitivity, and not due to “FGF21 resistance” ^5^. Hence, the existence of “FGF21 resistance” during obesity has remained controversial with the prevailing question: how can “FGF21 resistance” exist if pharmacological dosing is still efficacious ^1^? Potentially, FGF21 sensitivity during obesity may be akin to insulin resistance whereby the biological effect of endogenous FGF21 is lacking yet pharmacological dosing elicits an effect. Therefore, although effects of exogenous FGF21 should be evaluated in testing FGF21 sensitivity ^4^, consideration of the dose, functional readout, and time-course of FGF21 action should be taken.

Although plasma FGF21 levels, FGF21 co-receptor expression and downstream signaling should be evaluated in defining “FGF21 resistance” ^4^, it is difficult to interpret tissue-specific decreases in receptor and signaling activation without understanding how that specific tissue mediates FGF21’s effects. For example, what is the relevance of decreased co-receptor expression in white adipose tissue? Adipose tissue is necessary for FGF21’s acute insulin sensitizing effect ^6^ yet, different results have been reported *in vivo* when either overexpressing or maintaining physiological levels of β-klotho during obesity ^7, 8^. Furthermore, brown adipocytes mediate the acute insulin sensitizing action of FGF21 ^6^; meaning, that although decreased white adipose co-receptor expression has been used as a marker of “FGF21 resistance” we still do not understand the pathological relevance of this event.

How then, should “FGF21 resistance” during obesity be defined? Different experimental designs are required when evaluating the acute insulin sensitizing action of physiological levels of FGF21 versus the chronic effects on body composition of pharmacological doses of FGF21. Acute FGF21 sensitivity should be determined via insulin tolerance test following co-injection of insulin and FGF21 in addition to assessing tissue specific glucose uptake and activation of the FGF21 signaling cascade in brown adipose tissues ^6^. To test chronic FGF21 sensitivity, weight loss and energy expenditure should be evaluated, although FGF21 signaling is difficult to assess since the specific tissue(s) mediating these effects of chronic FGF21 treatment remain undetermined.

 Finally, caution is warranted in translating rodent studies to man. Although plasma FGF21 is elevated in obese and diabetic humans ^9^, human FGF21 is proteolytically cleaved *in vivo* ^10^. Therefore, the bioactivity of increased circulating FGF21 in humans remains unknown. It is possible that increased circulating FGF21 during obesity could serve a yet uncharacterized role. FGF21 has been shown to have central effects ^11– 13^, however whether or not central FGF21 co-receptor expression and signaling are altered during obesity remains unreported.

There is still much to discover regarding FGF21 action but consideration of the points outlined here can help avoid ambiguity in defining “FGF21 resistance” during obesity. Undoubtedly, the definition of “FGF21 resistance” will continue to evolve as new physiological and pharmacological studies help unravel the mechanisms underlying FGF21’s metabolic actions.

## Data availability

*All data underlying the results are available as part of the article and no additional source data are required*
