# Supplementary material for: Defining “FGF21 Resistance” during obesity: Controversy, criteria and unresolved questions
Source: F1000Res. 2018 Mar 7;7:289. [Version 1] doi: 10.12688/f1000research.14117.1 (PMC6020717; doi:10.12688/f1000research.14117.1)
Supplement: Peer review history

# Peer review: Referee response for version 1

**DOI:** 10.5256/f1000research.15354.r34679

**Published:** 2018-06-26

**Authors:** Andrew C. Adams

**Affiliations:** 1 Lilly Research Laboratories, Eli Lilly and company, Indianapolis, IN, USA

The opinion presented by Dr. Markan discusses an important topic in the FGF21 field, which has persisted as an unresolved issue for quite some time. While the report presents a balanced view, I would suggest the following potential additions/revisions:

- In the context of a discussion of FGF21 resistance and proteolytic cleavage, it is important to discuss recent developments as they pertain to the various circulating forms of FGF21. Specifically, it would be warranted to mention that it has been recently demonstrated that the ratio of ‘active’ FGF21 to total FGF21 can be modulated in humans (for example, in the GTT setting) and that in certain disease states, expression of the protease FAP are altered.
- It would be helpful to mention the composition of the FGF21 receptor complex early in the manuscript, prior to discussion of tissue specific effects later in the article. Indeed, it is possible that there is local FGF21 resistance in specific tissues, as measured by pERK vs. traditional systemic hormonal resistance.
- When discussing translation of FGF21 results to man, it is important to consider that many of the proposed clinical candidates in this area have the site of FAP cleavage mutated, thus negating C terminal truncation (likely the most dramatic inactivation by endogenous proteases). While N terminal cleavage may still occur, it is likely that truncation would not impair action, unless the treatment was with wild type human FGF21.
- Inclusion of discussion of more current findings from numerous groups on tissue specific ablation/overexpression of FGF21 receptor components would add significantly to the manuscript, specifically, detailed discussion of central vs. peripheral action might be relevant to the topic of ‘FGF21 Resistance’.
- Another potential explanation for increased FGF21 in states such as obesity is that it may be a sustained homeostatic response to chronic insult. Indeed, FGF21 appears is elevated by a number of stressors (both acute and chronic), including oxidative stress, lipopolysaccharide (LPS), ethanol/alcohol and dietary stress (Fructose etc). Interestingly, when these stressors are removed, FGF21 levels typically normalize relatively rapidly.

I have read this submission. I believe that I have an appropriate level of expertise to confirm that it is of an acceptable scientific standard, however I have significant reservations, as outlined above.

# Peer review: Referee response for version 1

**DOI:** 10.5256/f1000research.15354.r34680

**Published:** 2018-06-08

**Authors:** Junichiro Sonoda

**Affiliations:** 1 Department of Cancer Immunology, Genentech Inc., South San Francisco, CA, USA

Markan KR discusses the controversial “FGF21 resistance” in obesity. It is an important topic for discussion given the crucial role of FGF21 signaling in health and disease. However, I feel that the discussion should also incorporate more recent findings, especially the results of tissue-specific CKO studies which have shed light on the sites of FGF21 action (instead of just stating “the specific tissue(s) mediating these effects of chronic FGF21 treatment remain undetermined”). Also, highlighting examples of local FGF21 resistance (e.g., pERK as a marker) vs. systemic FGF21 resistance (e.g., whole body metabolic effects) would be necessary since the receptors for FGF21 are located in various cell types.    

 When the notion of “FGF21 resistance” was first introduced, it was an attractive idea to explain the “paradoxical” elevation in plasma FGF21 in obesity by analogy to other more established examples of hormone resistance where hormone insensitivity is associated with a feedback hormone production (e.g., insulin resistance, growth hormone resistance, leptin resistance, thyroid hormone resistance etc.). Around that time, the site of receptor expression important for the metabolic action of FGF21 was poorly understood, but the receptor complex expressed in adipocytes was thought to be responsible for many of the chronic metabolic effects. A decrease in Beta-Klotho protein expression in white adipose tissues in obese mice was thus considered to be a potential mechanism for “FGF21 resistance”. More recently, mouse genetic studies have uncovered that the induction of weight loss, glucose lowering, stimulation of energy expenditure, and increased water consumption, are mediated by the receptor complex expressed in the nervous system, rather than adipocytes (Owen et al. 2014 Cell Metab, etc.). This notion is completely overlooked by Markan KR, but in my view, is important when discussing “FGF21 resistance”.

 Other comments:

- Beta-Klotho was introduced in the 3rd paragraph without an explanation. For readers who are not familiar with FGF21 signaling, it should be explicitly noted that FGF21 acts by activating membrane-bound FGFR/Beta-Klotho receptor complex.
- In the second paragraph, “blunted ERK phosphorylation” – which tissues were examined?

I have read this submission. I believe that I have an appropriate level of expertise to confirm that it is of an acceptable scientific standard, however I have significant reservations, as outlined above.

# Peer review: Referee response for version 1

**DOI:** 10.5256/f1000research.15354.r31641

**Published:** 2018-03-27

**Authors:** Moosa Mohammadi

**Affiliations:** 1 Department of Biochemistry & Molecular Pharmacology, New York University School of Medicine, New York, NY, USA

In this article, Dr. Markan expresses her opinion on the ongoing controversy surrounding FGF21 resistance in obesity. This issue has not been explicitly addressed in the past publications so the commentary piece should be a refreshing read for the researchers in FGF signaling field in particular and obesity in general. Having said so, I recommend revising the text along the points below: 

 1) In the Abstract, I suggest changing the verb “used” to “introduced”. 

 2) First paragraph: I am wondering if the word “paradoxical” is the appropriate term to use here. Surely one could argue that the observed increases in FGF21 serum level in obese mice/human subjects represent a natural feedback mechanism in response to the progressive worsening of insulin resistance.  Are there any published literature which correlate the severity of obesity with serum levels of FGF21? If there are, they should be discussed.

 3) Clearly articulate/elaborate the difference between the two studies under scrutiny by putting particular emphasis on the different interpretations of dose response curves of FGF21 in these two studies. Also, please discuss how differences in experimental approach might possibly have influenced the disparate conclusions reached by the authors of these two studies. Explain whether only b-klotho co-receptor or both b-klotho and FGFR1c show reduced expression in obesity. Clearly identify tissues that lose expression of b-Klotho and/or FGFR1c and provide information on the extent of these expression losses if known.

 4) For the sake of general readership, please introduce b-Klotho co-receptor and FGFR1c, the cognate receptor of FGF21, early on in the text perhaps even in the abstract. Please state that unlike classical paracrine FGFs, FGF21 operates through a dual receptor system. 

 5) The point brought up on the inactivating proteolytic cleavage of FGF21 is interesting and worthy of further discussion. Please identify whether the reported increases in FGF21 levels measure the levels of full length bioactive form. I.e. are the assays used capable to differentiating between full length “active” and cleaved “inactive” forms.

I have read this submission. I believe that I have an appropriate level of expertise to confirm that it is of an acceptable scientific standard.
